# DNA repair protein RAD52 is required for protecting G-quadruplexes in mammalian cells

**DOI:** 10.1016/j.jbc.2022.102770

**Published:** 2022-12-05

**Authors:** Shuo Liu, Zi Wang, Sameer Bikram Shah, Chia-Yu Chang, Michael Ai, Tran Nguyen, Rong Xiang, Xiaohua Wu

**Affiliations:** 1 Department of Molecular Medicine, The Scripps Research Institute, La Jolla, California, USA; 2 School of Medicine, Nankai University, Tianjin, China

**Keywords:** RAD52, XPF, G-quadruplex, homologous recombination (HR), FANCJ, synthetic lethality, CFS, common fragile site, CFS-AT, CFS-derived AT-rich sequences, ChIP, chromatin immunoprecipitation, DSB, double-strand breaks, EGFP, enhanced GFP, G4, G-quadruplexes, HR, homologous recombination, PDS, pyridostatin, RPA, replication protein A

## Abstract

G-quadruplex (G4)-forming DNA sequences are abundant in the human genome, and they are hot spots for inducing DNA double-strand breaks (DSBs) and genome instability. The mechanisms involved in protecting G4s and maintaining genome stability have not been fully elucidated. Here, we demonstrated that RAD52 plays an important role in suppressing DSB accumulation at G4s, and RAD52-deficient cells are sensitive to G4-stabilizing compounds. Mechanistically, we showed that RAD52 is required for efficient homologous recombination repair at G4s, likely due to its function in recruiting structure-specific endonuclease XPF to remove G4 structures at DSB ends. We also demonstrated that upon G4 stabilization, endonuclease MUS81 mediates cleavage of stalled replication forks at G4s. The resulting DSBs recruit RAD52 and XPF to G4s for processing DSB ends to facilitate homologous recombination repair. Loss of RAD52 along with G4-resolving helicase FANCJ leads to a significant increase of DSB accumulation before and after treatment with the G4-stabilizing compound pyridostatin, and RAD52 exhibits a synthetic lethal interaction with FANCJ. Collectively, our findings reveal a new role of RAD52 in protecting G4 integrity and provide insights for new cancer treatment strategies.

Homologous recombination (HR) plays an important role in the maintenance of genome stability (1). In yeast, the *RAD52* epistasis group, including *RAD51*, *RAD52* itself, and many other proteins, is required for HR (2). HR is initiated by end resection and the resulted ssDNA is bound by replication protein A (RPA) (3). Yeast Rad52 plays an important role in promoting Rad51 replacement of RPA to form nucleoprotein filaments for homologous DNA search and strand exchange (4). In mammalian cells, although RAD52 still interacts with RAD51 and RPA (5, 6), the RAD51 mediator role is mainly carried out by BRCA2 but not RAD52 (7, 8), and hence mammalian RAD52 is dispensable for HR (9). On the other hand, different from the RAD51 mediator activity, RAD52 strand-annealing activity is conversed among different species and RAD52 is required for single-strand annealing in mammalian cells (10, 11, 12).

While yeast Rad52 is critical for most recombination processes (4), loss of RAD52 in mammalian cells does not show strong DNA repair defects and *RAD52* KO mouse lacks obvious phenotypes (13). However, more recently, accumulating evidence has revealed multiple new roles of RAD52 in the maintenance of genome stability in vertebrates. Loss of RAD52 results in the synthetic lethality of cells defective in a number of genes important for HR, such as BRCA1, BRCA2, PALB2, and RAD51 paralogs, suggesting a backup role of RAD52 in HR (14, 15, 16, 17). RAD52 binds to stalled replication forks and prevents excessive replication fork reversal, thereby protecting forks from unscheduled degradation (18). RAD52 is involved in promoting repair-mediated DNA synthesis following replication stress, likely through the break-induced replication pathway (19, 20). RAD52 also plays important roles in supporting the alternating length of telomeres (21, 22, 23, 24, 25) and in resolving R-loops and promoting transcription-associated HR (26, 27). In our previous study, we discovered that RAD52 is required for preventing double-strand break (DSB) accumulation at common fragile sites (CFSs) (28). Although RAD52 is not important for general HR in mammalian cells, it becomes indispensable for HR when DSB ends contain structure-prone AT-rich DNA sequences derived from CFSs, but the underlying mechanism is not clear.

Besides AT-rich sequences from CFSs, G-rich ssDNA could also adopt DNA secondary structures, known as G-quadruplexes (G4s), which are four-stranded helical DNA structures with four guanines arranged within a planar quartet (29). G4s are the hot spots to induce DNA damage and genome instability and are often found at chromosomal rearrangement sites in cancer (30). More than 700,000 sequences are detected to have the potential to form G4s in the human genome (31). G4s have important regulatory roles for different biological activities and often located in promoters, untranslated regions of mRNA, telomeres, and replication origins (32). The HR pathway has been shown to be important for repairing G4-induced DNA damage, and both BRCA1- and BRCA2-deficient cells are sensitive to G4-stabilizing compounds (33).

In this study, we demonstrated that RAD52 plays an important role in protecting G4s in mammalian cells. Deficiency in RAD52 leads to DSB accumulation upon treatment of G4-stabilizing compounds, and RAD52-deficient cells are sensitive to G4-stabilizing compounds. We further showed that RAD52 is required for recruiting structure-specific endonuclease XPF (34) to G4s to process DSB ends containing G4s, which is important for efficient HR. We also demonstrated that RAD52 is synthetically lethal with FANCJ, and this is consistent with the role of FANCJ in resolving G4s (35) to prevent DSB formation and the function of RAD52 in processing G4s with XPF at broken G4s to promote HR repair.

## Results

### RAD52-deficient cells are sensitive to G4-stabilizing drugs

**Figure 1:**
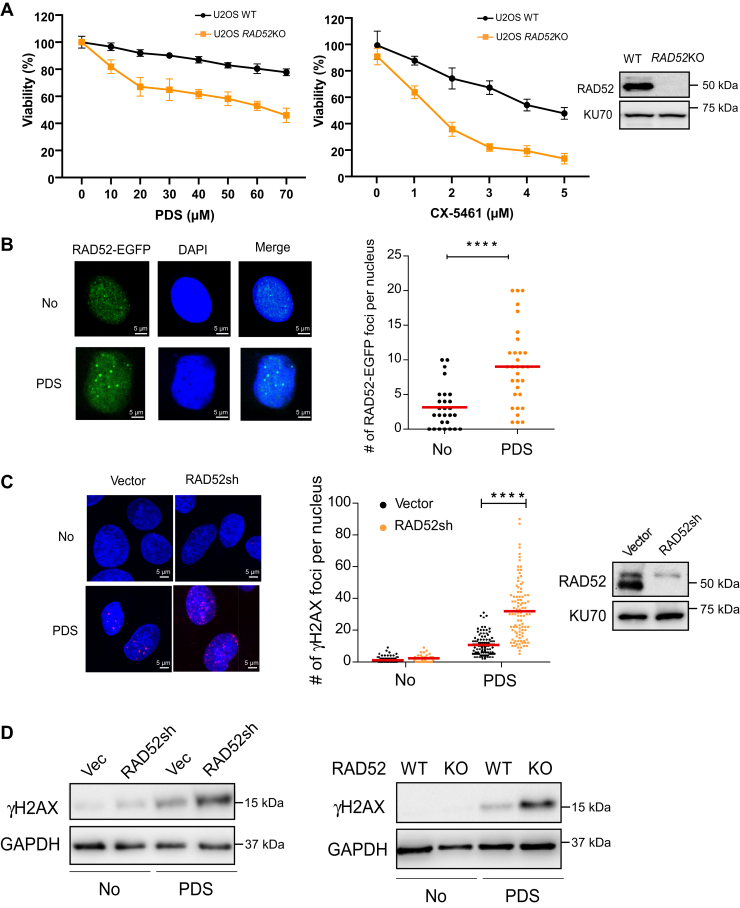
**RAD52 deficiency causes increased DSB formation and cell death after the treatment of G4-stabilizing drugs.***A*, U2OS and U2OS-derived *RAD52* KO cells were treated with the indicated concentrations of PDS (*left*) and CX-5461 (*middle*) for 48 hours (h), and cell viability assays were performed. RAD52 Western blot was performed to show *RAD52* KO with KU70 as a loading control (*right*). *B*, EGFP-RAD52 was expressed in U2OS cells and representative EGFP-RAD52 foci are shown with DAPI staining before and after PDS treatment (50 μM, 48 h, *left*). EGFP-RAD52 foci/nucleus in PDS-treated and -untreated cells were qualified and plotted (*right*). The *p* value is indicated as ∗∗∗∗*p* < 0.0001. *C*, representative γ-H2AX foci are shown in control (*vector*) and RAD52 shRNA-expressing U2OS cells treated or untreated with 50 μM PDS for 48 h (*left*). γ-H2AX foci/nucleus in PDS-treated and -untreated cells were qualified and plotted (*middle*). The *p* value is indicated as ∗∗∗∗*p* < 0.0001. RAD52 Western blot was performed to show RAD52 depletion by shRNA with KU70 as a loading control (*right*). *D*, U2OS cells expressing vector or shRNA for RAD52 (*left*) and U2OS and U2OS-derived *RAD52* KO cells (*right*) were treated with or without PDS (50 μM, 24 h), followed by γ-H2AX Western blot analysis using GAPDH as the loading control. DAPI, 4′,6-diamidino-2-phenylindole; DSB, double-stranded break; EGFP, enhanced GFP; G4, G-quadruplexes; PDS, pyridostatin.

RAD52 is important for protecting structure-prone AT-rich sequences derived from CFSs (28). To test whether RAD52 is also involved in maintaining G4 integrity, we generated *RAD52* KO U2OS cells and exposed them to the G4-stabilizing compounds pyridostatin (PDS) and CX-5461 (36). Compared to WT cells, the viability of *RAD52* KO cells after PDS and CX-5461 treatment is significantly reduced (Fig. 1*A*). We also demonstrated that PDS treatment induces more RAD52 foci as shown by expressing enhanced GFP (EGFP)-fused RAD52 (Fig. 1*B*). Depletion of RAD52 by shRNA leads to increased γH2AX foci formation (Fig. 1*C*). Similarly, RAD52 depletion and *RAD52* KO showed increased γH2AX signals after PDS treatment as revealed by Western blot analysis (Fig. 1*D*). These data suggest that RAD52 prevents DSB accumulation and protects cell viability when G4 structures are stabilized by G4-stabilizing compounds.

### RAD52 is specifically required for HR-mediated repair at DSB ends carrying G4s

**Figure 2:**
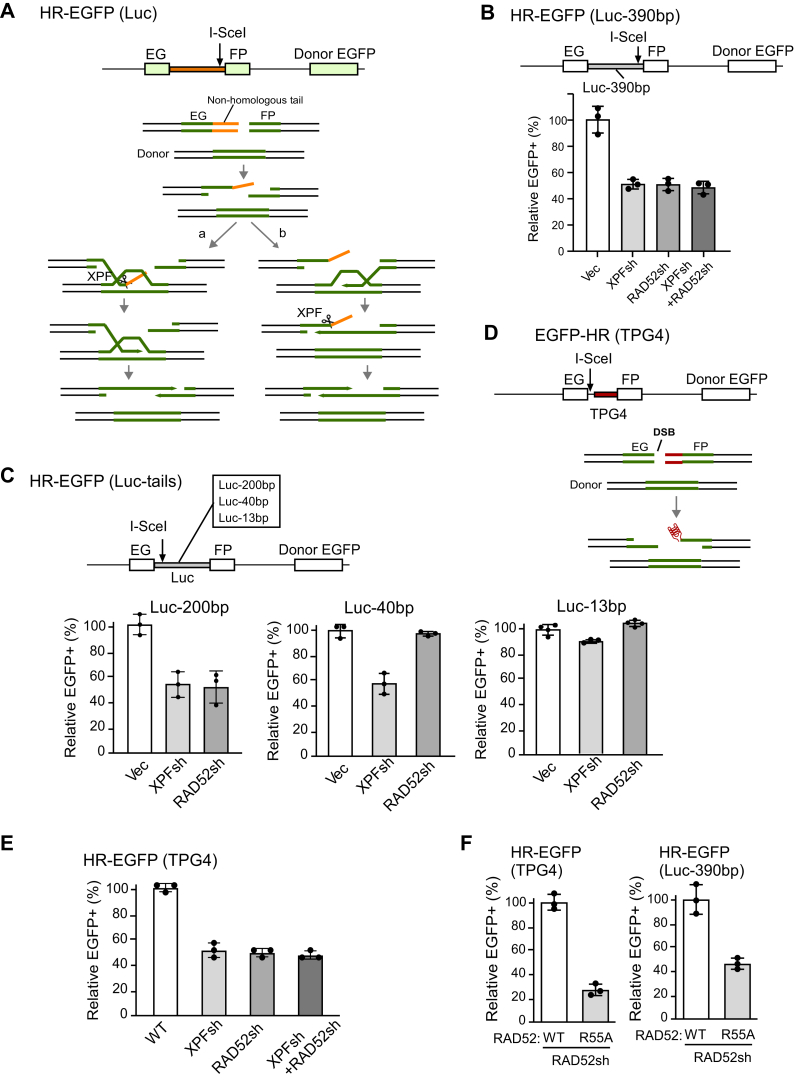
**RAD52 is required for HR when long nonhomologous tails or G4s are present at the DSB ends after I-SceI cleavage.***A*, schematic drawing of the HR-EGFP reporter containing a nonhomologous sequence (*orange*) in the recipient cassette serving as a nonhomologous tail after I-SceI cleavage (*top*). Models of HR repair at DSB ends containing nonhomologous tails (*bottom*). XPF/ERCC1 cleaves nonhomologous tails after strand invasion (a, *left*) or after second-end capture (b, *right*). *B*, U2OS cells carrying the HR-EGFP (Luc-390bp) reporter were depleted for XPF, RAD52, or both XPF and RAD52 by shRNAs with vector as control. Relative HR frequency was determined by FACS analysis 4 days after infection with lentiviruses producing I-SceI. *C*, U2OS cells carrying the HR-EGFP reporters with different lengths of inserted luciferase sequences (Luc-200 bp, Luc-40 bp, and Luc-13 bp) were depleted for XPF or RAD52 by shRNAs with vector as control. Relative HR frequency was determined by FACS analysis 4 days after the lentiviral infection of I-SceI. *D*, schematic drawing of the HR-EGFP (TPG4) reporter with a 40 bp insertion containing the TPG4 sequence with the I-SceI cleavage sites indicated (*top*). Upon I-SceI cleavage, G4 structures would form on the ssDNA overhangs after end resection (*bottom*). *E*, U2OS cells carrying the HR-EGFP (TPG4) reporter were depleted for XPF, RAD52, or both XPF and RAD52 by shRNAs with vector as control. Relative HR frequency was determined by FACS analysis 4 days after the lentiviral infection of I-SceI. *F*, RAD52 WT allele or R55A mutant allele was expressed in U2OS [HR-EGFP (TPG4)] cells (*left*) and U2OS [HR-EGFP (Luc-390 bp)] cells (*right*) and the endogenous RAD52 was depleted by shRNA (the shRNA targeting site in RAD52-WT and R55A was mutated). The relative HR frequency was determined by FACS analysis 4 days after the lentiviral infection of I-SceI. In all experiments, error bars represent the SD of at least three independent experiments. DSB, double-stranded break; EGFP, enhanced GFP; FACS, fluorescence activated cell sorting; G4, G-quadruplexes; HR, homologous recombination.

We showed that in mammalian cells, RAD52 is dispensable for general HR while it is required for HR at DSBs containing structure-prone CFS-derived AT-rich sequences (CFS-ATs) at the ends (28), but the underlying mechanism has not been addressed. To initiate HR, the 3′ ssDNA invades the homologous template and starts DNA synthesis (2). However, when nonhomologous tails are present or DNA secondary structures are formed at the 3′ ssDNA overhangs (Fig. 2, *A* and *D*), they need to be removed prior to HR (37, 38) (Fig. 2, *A*). The nonhomologous tails or DNA secondary structures would prevent DNA synthesis from the invading strand (Fig. 2*A*a, left) or the second capturing end (Fig. 2*A*b, right) depending on which end is used for strand invasion. We showed that XPF is involved in removing DNA nonhomologous tails and DNA secondary structures (such as CFS-ATs and G4s) at DSB ends to promote HR (37). Since RAD52 and XPF/ERCC1 directly interact with each other (39), we asked whether RAD52 functions together with XPF for the removal of nonhomologous tails and G4s at DSB ends to promote HR.

We first depleted RAD52 or XPF by shRNA in the HR reporter containing a 390-bp luciferase (Luc) sequence between the EG and FP cassettes [EGFP-HR (Luc-390 bp), Figs. 2*B* and S1*A*]. After I-SceI cleavage, a long nonhomologous tail (390 bp) is present at the DSB end. Both RAD52 and XPF are required for HR in the EGFP-HR (Luc-390 bp) reporter. Depleting both RAD52 and XPF only slightly further reduces HR than depleting RAD52 or XPF alone. This suggests that RAD52 is epistatic with XPF for HR repair in the EGFP-HR (Luc-390 bp) reporter, and this is consistent with the notion that RAD52 supports XPF activity in removing nonhomologous tails to promote HR.

We then established several additional HR reporters containing different lengths of the Luc sequences, and after I-SceI cleavage, these reporters would produce different lengths of nonhomologous tails (Fig. 2*C* top). Consistent with our previous findings that XPF is largely required for HR when nonhomologous tails are longer than 20 bp (37, 38), depleting XPF significantly reduces HR when nonhomologous tails are 200 bp and 40 bp in length but has a minor effect when the tail is 13 bp long (Figs. 2*C* and S1*B*). However, when we depleted RAD52 by shRNAs, we observed a decrease in HR when nonhomologous tails are 200 bp, but not 40 bp and 13 bp (Figs. 2*C* and S1*B*). These data suggest that RAD52 is only required to facilitate XPF to remove long but not short (≤40 bp) nonhomologous tails.

To test whether RAD52 is also needed to support XPF to remove G4 structures at DSBs to facilitate HR, we inserted a 31-bp G4 motif (TPG4) derived from mouse immunoglobulin locus (40) to our HR reporter at the side of I-SceI (Fig. 2*D*, top). After I-SceI cleavage, a 40-bp nonhomologous tail would be generated at the DSB ends, which contains the G4 motif and a part of the I-SceI cleavage sequence. After end resection, the G4 structure would form on the ssDNA overhang (Fig. 2*D*). Interestingly, we found that when DSBs contain the G4 motif (TPG4), 40 bp in length, both RAD52 and XPF are required for HR (Figs. 2*E* and S1*C*), although only XPF but not RAD52 is required for HR when a nonhomologous Luc-40 bp is present at the DSB ends (Fig. 2*C*, middle). We also showed that depleting both RAD52 and XPF does not cause any significant further decrease in HR than single depletion as assayed in the EGFP-HR (TPG4) reporter (Figs. 2*E* and S1*C*). These data suggest that RAD52 is required for HR when short nonhomologous tails contain G4 structures, although it is not needed to support XPF for removing short nonhomologous tails (≤40 bp). The epistatic relationship of RAD52 and XPF in the assay using the EGFP-HR (TPG4) reporter is consistent with the notion that RAD52 supports XPF to process G4 to promote HR.

We also expressed shRNA-resistant RAD52 WT allele and R55A mutant that is defective in ssDNA binding (41, 42) in U2OS cells with endogenous RAD52 depleted by shRNA. We found that HR is significantly impaired in the RAD52-R55A mutant when the DSB ends contain the G4 motif (TPG4) (Fig. 2*F*, left and Fig. S1*D*) or long nonhomologous tail (Luc-390 bp) (Fig. 2*F*, right and Fig. S1*D*). These data suggest that RAD52 ssDNA-binding activity is important for RAD52 to facilitate XPF-mediated removal of 3′ G4 structures or long nonhomologous tails.

### G4 structures induce mitotic recombination upon PDS treatment or FANCJ loss in a manner dependent on MUS81 and RAD52

**Figure 3:**
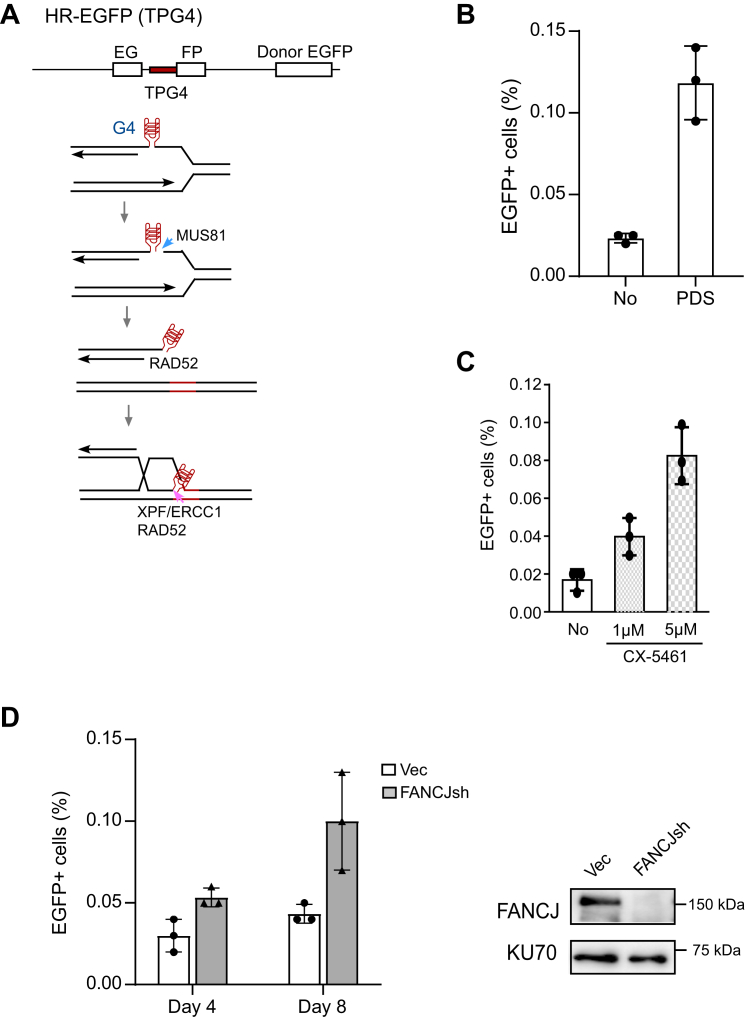
**G4-stabilizing drugs and FANCJ deficiency induce mitotic recombination at G4s.***A*, schematic drawing of the model to show fork stalling, fork breakage, and HR repair at G4 sites. *B* and *C*, U2OS [HR-EGFP (TPG4)] cells treated with PDS (50 μM, 72 h) (*B*) and indicated concentrations of CX-5461 (72 h) (*C*) or without (No), and mitotic recombination was determined by FACS analysis of EGFP-positive cells. *D*, U2OS [HR-EGFP (TPG4)] cells were depleted for FANCJ by shRNA using vector (Vec) as control, and mitotic recombination was determined by FACS analysis 4 or 8 days after infection of FANCJ shRNA lentiviruses. FANCJ depletion is shown by Western blot with KU70 as a loading control. In all experiments, error bars represent the SD of at least three independent experiments. EGFP, enhanced GFP; FACS, fluorescence activated cell sorting; G4, G-quadruplexes; HR, homologous recombination; PDS, pyridostatin.

When G4 structures are stabilized or accumulated in the genome, they induce DSB formation (43). This is likely due to fork stalling and subsequent breakage at G4s (Fig. 3*A*). To monitor whether HR could be used to repair DSBs induced by G4s, we determined mitotic recombination using the EGFP-HR (TPG4) reporter that contains a G4 motif (Fig. 3*A*). Indeed, PDS or CX-5461 treatment leads to increased spontaneous HR (Fig. 3, *B* and *C*). FANCJ is a helicase to unwind G4s (44, 45), thereby preventing G4 structure formation. We depleted FANCJ by shRNA and found that spontaneous HR is also significantly increased at G4s in the EGFP-HR (TPG4) reporter (Fig. 3*D*). These data suggest that G4-induced DSBs can be repaired by HR.

**Figure 4:**
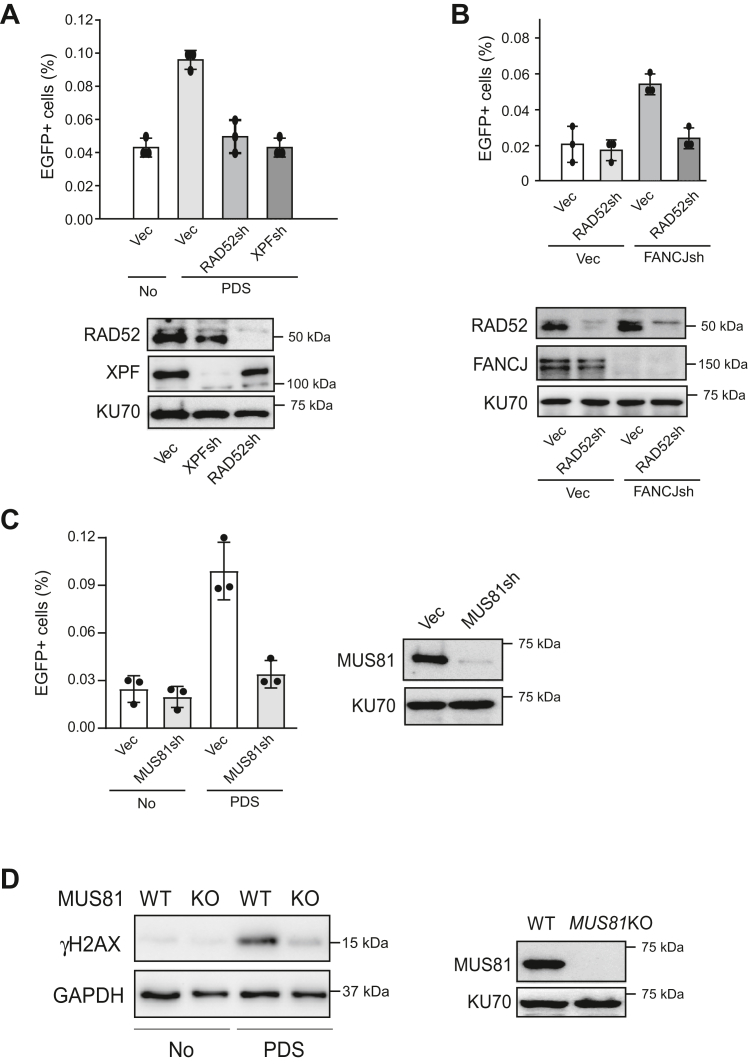
**Mitotic recombination at G4s induced by G4-stabilizing drugs or FANCJ deficiency is RAD52-dependent.***A*, U2OS [HR-EGFP (TPG4)] cells were depleted for XPF or RAD52 by shRNAs with vector (Vec) as control. Mitotic recombination frequency was determined by FACS analysis before (No) and after PDS (50 μM, 72 h) treatment (*top*). RAD52 and XPF depletion is shown by Western blot with KU70 as a loading control (*bottom*). *B*, U2OS [HR-EGFP (TPG4)] cells were depleted for RAD52 by shRNAs using vector (Vec) as the control, followed by depleting FANCJ with shRNAs or expressing shRNA vector (Vec). Mitotic recombination frequency was determined by FACS analysis 4 days after infection of FANCJ shRNA lentiviruses (*top*). RAD52 and FANCJ depletion is shown by Western blot with KU70 as a loading control (*bottom*). *C*, U2OS [HR-EGFP (TPG4)] cells were depleted for MUS81 by shRNAs with vector (Vec) as the control. Mitotic recombination frequency was determined by FACS analysis before (No) and after PDS (50 μM, 72 h) treatment (*left*). MUS81 depletion is shown by Western blot with KU70 as a loading control (*right*). *D*, U2OS and *MUS81*KO U2OS cells were treated with or without PDS (50 μM, 48 h), followed by γ-H2AX Western blot analysis using GAPDH as the loading control (*left*). MUS81 Western blot with KU70 as a loading control is present to show *MUS81*KO (*right*). In all experiments, error bars represent the SD of at least three independent experiments. EGFP, enhanced GFP; FACS, fluorescence activated cell sorting; G4, G-quadruplexes; HR, homologous recombination; PDS, pyridostatin.

To examine whether RAD52 is also needed for HR to repair DSBs induced by G4s, we depleted RAD52 by shRNAs in the EGFP-HR (TPG4) reporter cell line. PDS-induced mitotic recombination is much reduced when RAD52 shRNA is expressed, to the extent comparable to that when XPF is depleted (Fig. 4*A*). Similarly, mitotic recombination at G4s induced by FANCJ depletion is also dependent on RAD52 (Fig. 4*B*).

We propose that when replication forks are stalled at G4s, MUS81 cleaves stalled forks, resulting in DSB formation (Fig. 3*A*). Indeed, PDS-induced mitotic recombination at G4s is significantly reduced when MUS81 is depleted by shRNA (Fig. 4*C*). We also showed that PDS-induced DSB accumulation in MUS81KO cells is much reduced as revealed by γH2AX Western blot analysis (Fig. 4*D*). These data support the model that MUS81 is responsible for generating DSBs at G4s on replication forks when the G4 structures are stabilized.

### RAD52 is required for XPF recruitment to G4s after DSB formation

**Figure 5:**
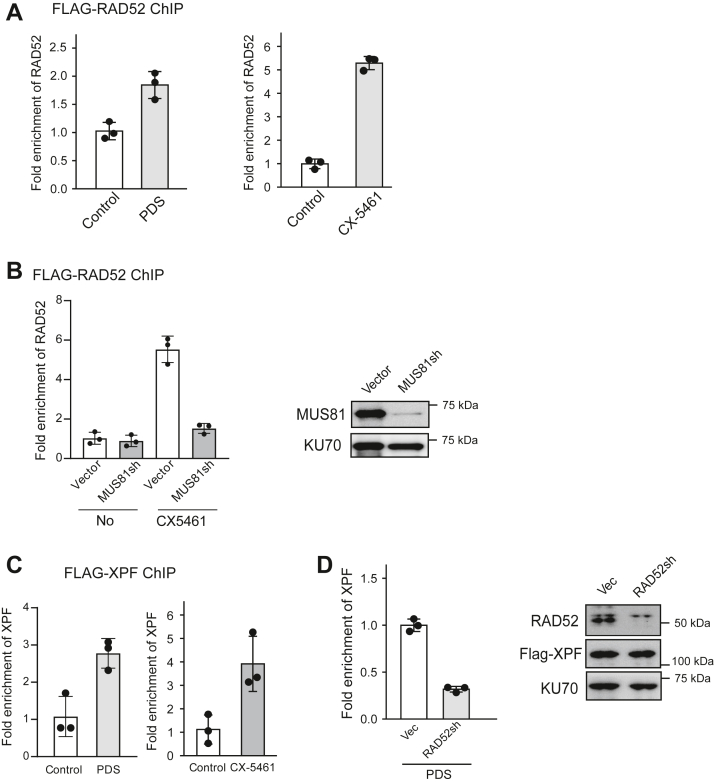
**XPF recruitment to G4 is dependent on RAD52.***A*, anti-Flag ChIP analysis at G4 locus was performed in U2OS [HR-EGFP (TPG4)] cells expressing Flag-RAD52 before and after PDS (50 μM, 48 h) or CX-5461 (1 μM, 48 h) treatment. Enrichment of RAD52 at G4 was calculated using the ChIP value in PDS-untreated cells as 1 for normalization. *B*, U2OS [HR-EGFP (TPG4)] cells expressing Flag-RAD52 were depleted for MUS81 by shRNAs with vector (Vec) as the control. Anti-Flag ChIP analysis at G4 locus was performed before and after CX-5461 (1 μM, 48 h) treatment. Enrichment of XPF at G4 locus was calculated using the ChIP value in CX-5461–untreated cells with vector control as 1 for normalization (*left*). MUS81 depletion is shown by Western blot with KU70 as a loading control (*right*). *C*, anti-Flag ChIP analysis at G4 locus was performed in U2OS [HR-EGFP (TPG4)] cells expressing Flag-XPF before and after PDS (50 μM, 48 h) or CX-5461 (1 μM, 48 h) treatment. Enrichment of XPF at G4 was calculated using the ChIP value in untreated cells as 1 for normalization. *D*, anti-Flag ChIP analysis at G4 locus was performed in U2OS [HR-EGFP (TPG4)] cells expressing Flag-XPF and with or without depletion of RAD52 by shRNAs upon PDS (50 μM, 48 h) treatment. Enrichment of XPF at G4 was calculated using the ChIP value in the vector control as 1 for normalization (*left*). RAD52 depletion is shown by Western blot with KU70 as a loading control (*right*). In all experiments, error bars represent the SD of at least three independent experiments. ChIP, chromatin immunoprecipitation; EGFP, enhanced GFP; G4, G-quadruplexes; HR, homologous recombination; PDS, pyridostatin.

By chromatin immunoprecipitation (ChIP) analysis, we showed that RAD52 is accumulated to G4s in the EGFP-HR (TPG4) reporter after PDS and CX-5461 treatment (Figs. 5*A* and S2*A*). We further showed that RAD52 binding to G4s after CX-5461 treatment is strongly reduced when MUS81 is depleted (Fig. 5*B*), suggesting that RAD52 is recruited after DSBs are generated by MUS81 at G4s. In addition, ChIP analysis also showed that XPF is recruited to G4s after PDS and CX-5461 treatment (Figs. 5*C* and S2*B*) and the recruitment of XPF to G4s is compromised when RAD52 is depleted (Fig. 5*D*). These data suggest that RAD52 facilitates XPF recruitment to G4s to mediate the cleavage of G4s (Fig. 3*A*).

### RAD52 is synthetically lethal with FANCJ

**Figure 6:**
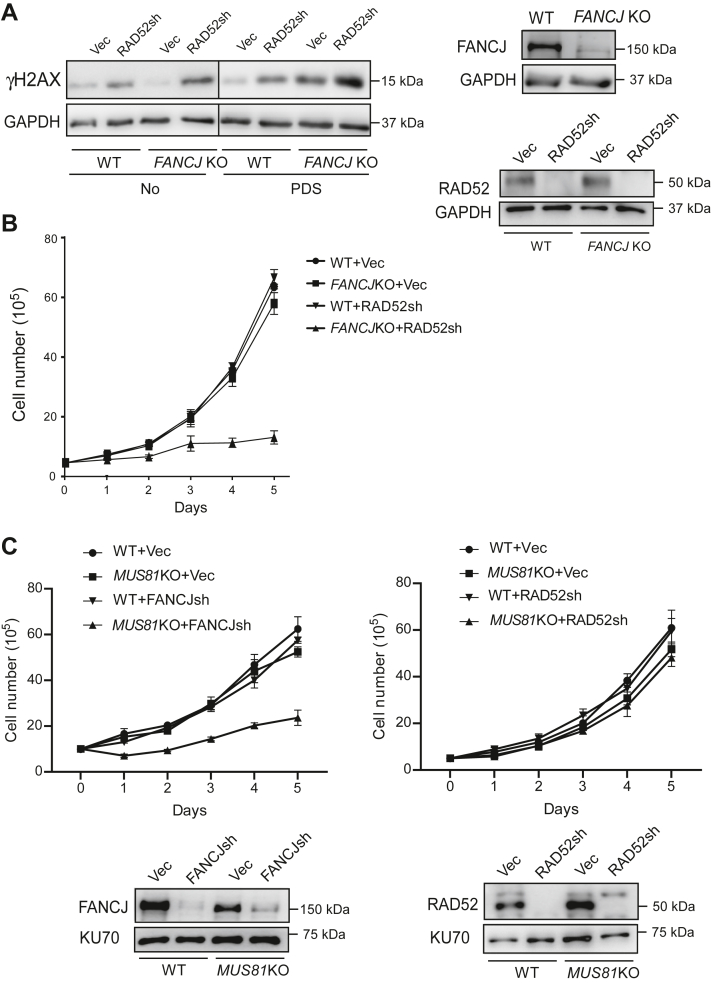
**RAD52 is synthetically lethal with FANCJ.***A*, U2OS cells were depleted for RAD52, FANCJ, or both by shRNAs with a vector control. γ-H2AX Western blot analysis was performed before and after PDS treatment (50 μM, 24 h) using GAPDH as the loading control (*left*). Depletion efficiency was determined by Western blot analysis using indicated antibodies (*right*). *B*, U2OS WT and *FANCJ* KO cells were infected with RAD52 shRNA lentiviruses or vector control, and cell growth curves were plotted (*left*). Error bars represent the SD of at least three independent experiments. RAD52 depletion and *FANCJ* KO were verified by Western blot analysis using indicated antibodies (*right*). *C*, U2OS WT and *MUS81*KO cells were infected with FANCJ shRNA (*left*) or RAD52 shRNA (*right*) lentiviruses or vector control, and cell growth curves were plotted (*top*). Error bars represent the SD of at least three independent experiments. Depletion of FANCJ and RAD52 was verified by Western blot analysis using indicated antibodies (*bottom*). PDS, pyridostatin.

Since FANCJ depletion causes an increase of HR at G4s in an RAD52-dependent manner, we examined γH2AX accumulation when FANCJ, RAD52, or both are depleted in the presence or absence of PDS treatment (Fig. 6*A*). Depletion of RAD52 significantly increases γH2AX levels in *FANCJ* KO cells without PDS treatment and with a further increase after PDS treatment. These data are consistent with the role of RAD52 in repairing DSBs at G4s that are accumulated due to loss of FANCJ. We also showed that depletion of RAD52 by shRNA in *FANCJ* KO cells drastically reduced the viability of *FANCJ* KO cells (Fig. 6*B*), suggesting that RAD52 is synthetically lethal with FANCJ.

We also examined whether MUS81-mediated cleavage of stalled replication forks at G4s is important for cell viability. *MUS81*KO does not impair cell viability, but depleting FANCJ by shRNA in *MUS81*KO cells leads to compromised cell growth (Fig. 6*C*, left). This suggests that when G4s are accumulated due to the loss of FANCJ, MUS81-mediated fork cleavage at accumulated G4s is important for recovering replication from stalled forks at G4s through DSB-repair mechanism. However, depletion of RAD52 in *MUS81*KO cells does not increase cell death (Fig. 6*C*, right). This is consistent with the model that RAD52 is involved in repairing DSBs after fork cleavage by MUS81 at G4s. Since G4s are not accumulated more in RAD52-deficient cells than in WT cells, it is not essential for MUS81 to cleave forks at G4s to promote replication recovery.

## Discussion

*RAD52* is not an essential gene in mammalian cells, and its function in HR is dispensable (9). However, further study revealed that RAD52 has an important backup role to BRCA2 in HR and also possesses multiple activities in coping with replication stress (9, 46). In this study, we identified a new role of RAD52 in protecting G4s and showed that RAD52-deficient cells are sensitive to G4-stabilizing drugs. We demonstrated that RAD52 is required for HR at the DSB ends that contain long nonhomologous tails or G4s.

Previously, we showed that RAD52 is required for HR when DSB ends contain CFS-ATs that form DNA secondary structures (28), but the underlying mechanism of how RAD52 is involved was not elucidated. In this study, we used G4s as a model system to analyze the mechanism for repairing DSBs with DNA secondary structures at the ends. We showed that the requirement of RAD52 for HR at DSBs carrying G4s is due to DNA secondary structure formation at G4s similar to that at CFS-ATs on DSB ends. We demonstrated that RAD52 is epistatic to XPF in HR when long nonhomologous DNA tails or G4s are present at DSBs after I-SceI cleavage, and the recruitment of XPF to G4s depends on RAD52 as revealed by ChIP analysis. Since RAD52 forms a complex with XPF and promotes XPF activity to remove FLAPs (39), we propose the model that RAD52 detects G4 or other DNA secondary structures and recruits XPF/ERCC1 through a physical interaction. It is possible that RAD52 binds to ssDNA nonhomologous tails or ssDNA adjacent to G4s (Fig. 3*A*) and recruits XPF–ERCC1 complex to FLAPs or G4s after strand annealing of the invading strands to the templates or second strand capture using internal homologous sequences (Fig. 2*A*). In addition, the binding of RAD52 with XPF may also stimulate XPF/ERCC1 activity to cleave nonhomologous tails in the form of FLAPs or DNA secondary structures. Previously, it was proposed that the role of RAD52 in promoting HR at DSBs containing DNA secondary structures could be due to the activity of RAD52 to assist RAD51 in initiating strand invasion from a blocked end using RAD52 ssDNA-annealing activity (28). Alternatively, the annealing activity of RAD52 may be important for second-end capture when the DSB ends are blocked (28). Although these proposed mechanisms remain possible, the epistatic genetic relationship of XPF and RAD52 supports the idea that RAD52 functioning together with XFP/ERCC1 in DSB end processing is likely the major mechanism for the requirement of RAD52 to repair DSBs with long nonhomologous or DNA secondary structures.

DNA secondary structures often induce replication stalling, causing DSB accumulation (47, 48, 49). MUS81 is important for cleaving stalled replication forks to generate DSBs, thus facilitating fork repair and replication restart (50). We showed that MUS81 is required for DSB formation in cells treated with G4-stabilizing drugs, suggesting that replication fork breakage is a major source for DSB formation at G4s and MUS81 is responsible for fork cleavage. We also showed that when G4s are accumulated due to the loss of FANCJ, MUS81 is important for maintaining cell viability. Hence, MUS81-mediated cleavage at stalled replication forks at G4s is an active process important for replication recovery at G4s through HR-mediated DSB repair. Since RAD52 recruitment to G4s depends on MUS81 upon treatment of G4-stabilizing drugs, RAD52 recruitment to G4s occurs after fork breakage and DSB formation. Collectively, the working model is that when G4s are stabilized or accumulated, replication forks are stalled, and MUS81 cleaves stalled replication forks, leading to DSB formation (Fig. 3*A*). After strand invasion, G4s at the DSB ends prevent DNA polymerases from accessing the 3′ ends to initiate HR-associated DNA synthesis. RAD52 binds to the ssDNA surrounding G4s and recruits XPF/ERCC1 to cleave G4s, thereby allowing repair DNA synthesis and subsequent HR steps.

FANCJ promotes the unwinding of G4s (44, 45). We showed that FANCJ deficiency indeed increases DSB accumulation and mitotic recombination at G4s after treatment of G4-stabilizing drugs. In addition, impaired function of RAD52 causes more DSB formation in FANCJ-deficient cells, which is further enhanced upon treatment of G4-stabilizing drugs. Thus, FANCJ and RAD52 in association with XPF/ERCC1 play concerted roles by removing G4s in our genome and repairing DSBs arising at G4s, respectively. Since G4s are so abundant in our genome, these two concerted functions of FANCJ and RAD52 underlie their genetically synthetic lethal interactions.

G4-stabilizing drugs have been used for cancer treatment (51, 52), and they exhibit more toxic effects in BRCA1/2-deficient and ATRX-deficient cancer cells (33, 53). Our study demonstrated that inactivation of RAD52 could sensitize cells to G4-stabilizing drugs, suggesting a new strategy to potentiate the effect of G4-stabilizing drugs. In addition, RAD52 is synthetically lethal with FANCJ, and inhibition of RAD52 causes more DSBs in FANCJ-deficient cells. RAD52 is not an essential gene in normal cells, and thus RAD52 is an optimal drug target for cancer treatment. Since FANCJ is a tumor suppressor and is associated with a broad range of cancers (54), inhibition of RAD52 in combination with G4-stabilizing drugs provides new strategies for targeted cancer treatment of FANCJ-deficient tumors.

## Experimental procedures

### Cell culture

U2OS and 293T cells were obtained from American Type Culture Collection and cultured at 37 °C and 5% CO_2_ in Dulbecco’s modified Eagle’s medium with 10% fetal bovine serum and 1% Penicillin-Streptomycin. 293T cells and U2OS cells were transfected using standard calcium phosphate protocol or Lipofectamine 2000 (Thermo Fisher Scientific). Infection of U2OS cells was performed using the standard lentiviral infection protocol.

### Plasmid construction and generation of repair reporter cell lines

Generation of Flag-tagged XPF was described previously (37). GFP-RAD52 is provided by Dr Kiyoshi Miyagawa (26). Flag-tagged RAD52 was constructed by inserting Flag tag and RAD52 cDNA into the pCDH-CMV-MCS-EF1-NEO vector. The Flag-RAD52-R55A mutant was generated by site-directed mutagenesis. Stable expression of Flag-RAD52-WT and R55A mutant and Flag-tagged XPF in U2OS cells were generated by lentiviral infection followed by G418 selection.

Luciferase fragments with different lengths (390 bp, 200 bp, and 40 bp) and TPG4 [a G4 substrate from the mouse immunoglobulin locus G4 sequences (40)] were inserted into the middle of EGFP recipient cassette on the HR-EGFP reporter described previously (49). TPG4 sequence: GGGGGAGCTGGGGTAGATGGGAATGTGAGGG. These reporters were transfected to U2OS cells, and after drug selection (hygromycin), single clones were picked and screened.

### Generation of *RAD52* KO, *MUS81*KO, and *FANCJ* KO cell lines by CRISPR

We obtained the sgRNA/Cas9 all-in-one vector from GeneCopoeia, Inc and inserted an mCherry marker into the vector for selection. To generate *RAD52*-KO, two gRNAs, gRNA3 (tccagaaggccctgaggcag) and gRNA4 (agtagccgcatggctggcgg), targeting the exon 3 of human *RAD52* were individually subcloned into the sgRNA/Cas9-mCherry vector. To generate *FANCJ*-KO, two gRNA/Cas9 plasmid constructs with gRNA1 (gattactagagagctccgg) and gRNA3 (gcacctagaacagtggccag) targeting exon 7 of the human *FANCJ* were used.

Two RAD52 gRNA/Cas9 plasmid constructs or two FANCJ gRNA/Cas9 plasmid constructs were transfected together to the target cell lines by PEI methods following the standard protocols. Forty eight hours after transfection, single clones were isolated by fluorescence activated cell sorting (FACS) of mCherry-positive cells. *RAD52*-KO clones and *FANCJ*-KO clones were screened by RAD52 and FANCJ Western blot analysis, respectively, and those without expression of the protein were further characterized by PCR of genomic DNA, followed by sequencing.

The construction of *MUS81-KO* was described previously (37).

### shRNA interference

Depletion of endogenous proteins was achieved by lentiviral infection using pLKO.1-puro vector (Addgene #8453) or pLKO.1-blast vector (Addgene #26655) to express corresponding shRNAs. shRNA target sequences are: RAD52 shRNA, gatgttggttatggtgttagt and ggatggttcatatcatgaaga; XPF shRNA, aagacgagctcacgagtattc; and FANCJ shRNA, gaacagaagtacacaatttgg.

### Immunoblotting

Western blot analysis was performed using the standard protocol as described previously (49). Cells were lysed in NETN buffer (20 mM Tris–HCl, pH 8.0, 100 mM NaCl, 0.5 mM EDTA, 0.5% NP-40) or RIPA buffer (for H2AX-S139p, 50 mM Tris–HCl, pH 8.0, 150 mM NaCl, 5 mM EDTA, 1% NP-40, 0.5% sodium deoxycholate, and 0.1% SDS) containing protease inhibitors, aprotinin (4 μg/μl) and PMSF (1 mM), and phosphatase inhibitors (Thermo Fisher Scientific, Cat# 88667). Cell lysates were boiled in 2 × SDS loading buffer and subjected to SDS-PAGE. Commercial antibodies used are as follows: XPF (ABclonal Science, Inc, A8119), MUS81 (Santa Cruz Biotechnology Inc, sc-376661), H2AX-S139p (Cell Signaling Technology, #2577), RAD52 (ABclonal Science, Inc, A5186), FANCJ (ABclonal Science, Inc, A6804), KU70 (Santa Cruz Biotechnology, Inc, sc-17789), and GAPDH (ABclonal Science, Inc, AC002).

### Immunofluorescence

Cells were seeded on coverslips and fixed with cold methanol for 15 min at −20 °C. Then, the cells were washed three times with PBST (PBS with 0.1% Tween-20), followed by blocking with 5% normal goat serum diluted by PBS. The primary antibodies were diluted by 5% normal goat serum to the appropriate concentrations and incubated with cells overnight at 4 °C. The slides were then washed with PBS with Tween-20 for three times and subsequently were incubated with diluted secondary antibodies at room temperature for 1 h. After washing and staining the nuclei with 4′,6-diamidino-2-phenylindole (DAPI), the slides were sealed with a quenching-preventive mounting medium. Images were recorded by a confocal microscope.

### Chromatin immunoprecipitation

ChIP was performed as described (28). Briefly, cultured cells were incubated with 1% formaldehyde for 10 min at room temperature and the crosslinking was then stopped by adding glycine to 0.125 M and incubating for 5 min. After washing with PBS for two times, cells were harvested and lysed in the lysis buffer (50 mM Tris–HCl, pH 8.1, 10 mM EDTA, 1% SDS, protease inhibitor cocktail complete) for 10 min on ice. Sonication was used to break chromatin DNA into fragments with an average length of about 0.4 kb. After centrifugation, the supernatant was precleared with Dynabeads Protein G (Invitrogen) and then incubated with anti-Flag M2 antibody (Sigma-Aldrich, F3165) and Dynabeads Protein G for overnight at 4 °C by rotation. The ChIP reactions were cleared by centrifugation and then washed with TSE I (0.1% SDS, 1% Triton X-100, 2 mM EDTA, 20 mM Tris–HCl, pH 8.1, and 150 mM NaCl), TSE II (0.1% SDS, 1% Triton X-100, 2 mM EDTA, 20 mM Tris–HCl, pH 8.1, and 500 mM NaCl), buffer III (0.25 M LiCl, 1% NP-40, 1% Sodium deoxycholate, 1 mM EDTA, 10 mM Tris–HCl, and pH 8.1), and TE buffer. The DNA–protein complex was eluted from beads by adding 120 μl elution buffer (1% SDS, 0.1 M NaHCO3) into the pellet of ChIP reactions. Then, 4 μl of 5 M NaCl was added to reverse DNA-protein crosslinking. After incubating at 65 °C for 6 h, proteinase K (2 μl of 20 mg/ml) was added and incubated at 42 °C for 2 h. DNA was purified from the reaction using the QIAquick kit (QIAGEN). The recovered DNA was analyzed by real-time PCR. The primers used for ChIP at TPG4 in the reporter cell line are as follows: F-AGCACGACTTCTTCAAGTCCG, R-AGGGTAATACCGGTCGCGC.

### Growth curve and cell viability assay

Cell proliferation was measured by hemocytometer counting of trypsinized cells every 24 h. Cell viability after drug treatment was determined by MTS assay (CellTiter 96 AQueous One Solution Cell Proliferation Assay, Promega Corporation). Briefly, cells were trypsinized and seeded in the 96-well plates (5000 cells/well), and 24 h later, cells were treated with different concentrations of PDS (Cayman Chemical Company, Cat#18013) or CX-5461 (Cayman Chemical Company, Cat#18392) for 48 h. After adding 10% MTS to each well, the cells were incubated at 37 °C for 2 h, and then the absorbance at A490 was read by a microplate reader (μQuant, BioTek) and normalized to the value of untreated cells.

### Quantification and statistical analysis

All data were analyzed using Prism GraphPad and Excel. In all experiments, error bars represent SD of at least three independent experiments. Student’s *t* test was performed to show statistical significance.

## Data availability

All data are included in the article.

## Supporting information

This article contains supporting information.

## Conflict of interest

The authors declare that they have no competing conflicts of interests.
