# Supplementary material for: DNA repair protein RAD52 is required for protecting G-quadruplexes in mammalian cells
Source: J Biol Chem. 2022 Dec 5;299(1):102770. doi: 10.1016/j.jbc.2022.102770 (PMC9807996; doi:10.1016/j.jbc.2022.102770)
Supplement: Supplemental Figures S1–S2 [file mmc1.pdf]

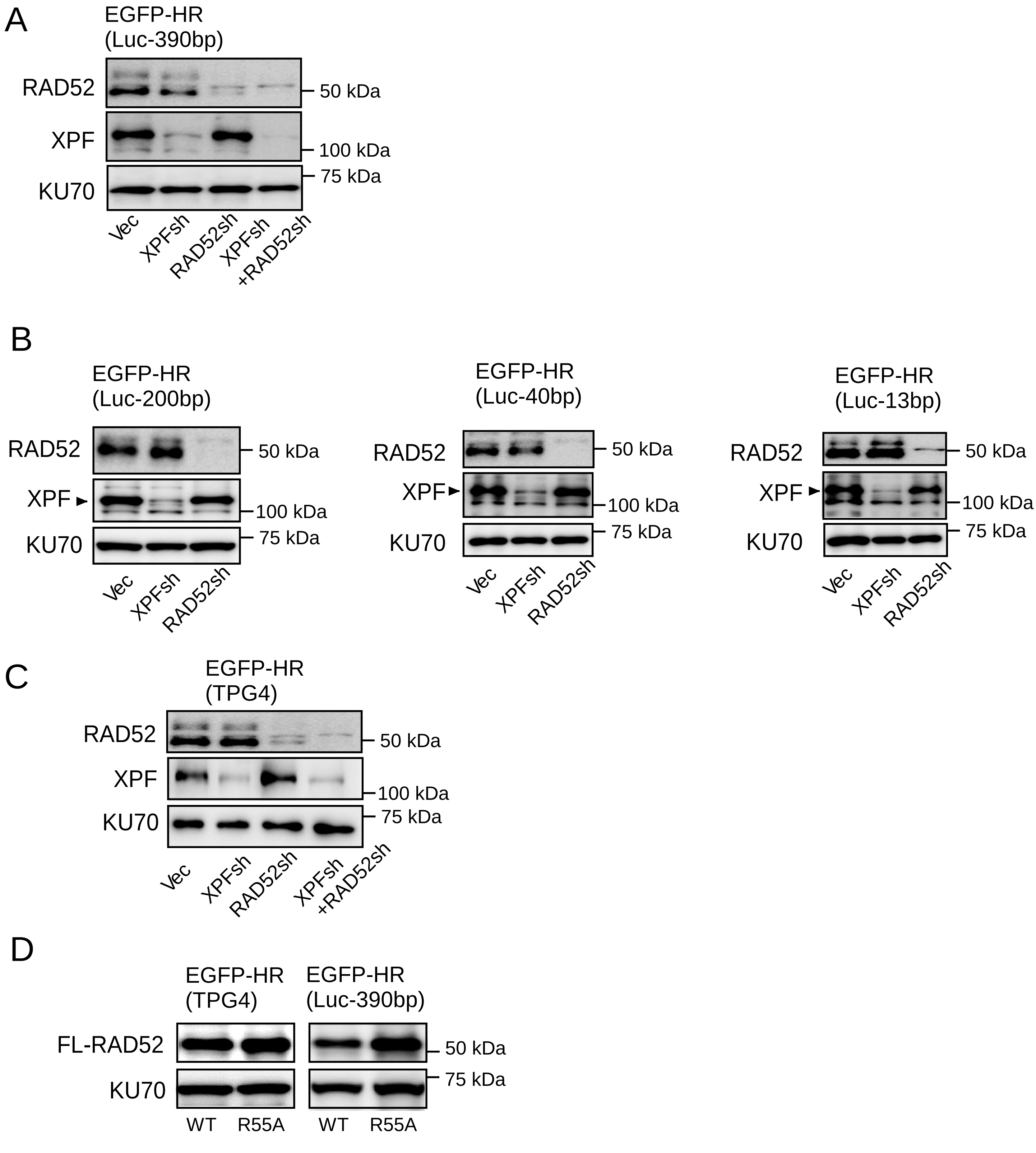

Figure S1. Western blot analysis to examine depletion of XPF and RAD52, and expression of Flag-RAD52WT and R55A mutant.

(A, B and C). Lentiviruses encoding shRNAs for XPF or RAD52 or vector were used to infect U2OS [EGFP-HR (Luc-390bp)] cells (A), U2OS [EGFP-HR (Luc-200bp), (Luc-40bp) and (Luc-13bp)] cells (B) and U2OS [EGFP-HR (TPG4)] cells (C). Western blot analysis was performed using indicated antibodies.

(D). Lentiviruses encoding Flag-RAD52-WT and R55A mutant were used to infect U2OS [EGFP-HR (TPG4)] and U2OS [EGFP-HR (Luc-390bp)] cells. Anti-Flag (M2) Western blotting was performed with KU70 as the loading control.

These cell lines were used in the experiments shown in Figure 2.

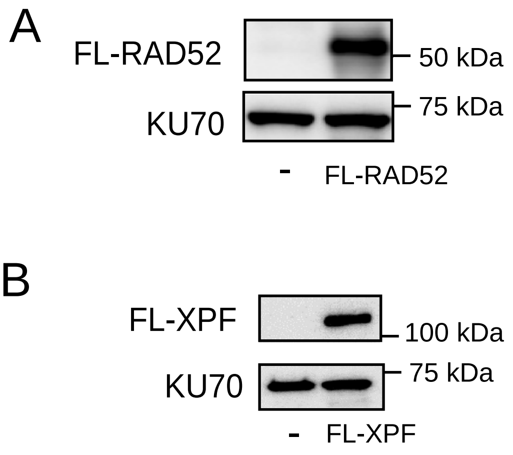

Figure S2. Generating U2OS [HR-EGFP (TPG4)] cell lines expressing Flag-RAD52 and Flag-XPF. Lentiviruses encoding Flag-RAD52 and Flag-XPF were used to infect U2OS [HR-EGFP (TPG4)] cells. After G418 selection, cells with or without Flag-RAD52 (A) and Flag-XPF (B) viral infection were lysed and subject to Western blotting using anti-Flag antibody (M2) with KU70 as the loading control. These cell lines were used in the ChIP analysis performed in Figure 5.
